# Progression to unscheduled hospital admissions in people with diabetes: a qualitative interview study

**DOI:** 10.3399/BJGPO.2021.0044

**Published:** 2021-06-02

**Authors:** Nikki ED Perrin, Janet Heaton, Sandra M MacRury, Kathleen M Friel, Vivien Coates

**Affiliations:** 1 Department of Psychology, University of the Highlands and Islands, Inverness, UK; 2 Division of Rural Health and Wellbeing, University of the Highlands and Islands, Inverness, UK; 3 Diabetes and Endocrinology, Raigmore Hospital, Inverness, UK; 4 Institute of Nursing Research, Ulster University, Londonderry, UK; 5 Nursing Directorate, Western Health and Social Care Trust, Londonderry, UK

**Keywords:** diabetes mellitus, prevention, qualitative research, emergency service, hospital

## Abstract

**Background:**

People with diabetes often have difficulty maintaining optimal blood glucose levels, risking progressive complications that can lead to unscheduled care. Unscheduled care can include attending emergency departments, ambulance callouts, out-of-hours care, and non-elective hospital admissions. A large proportion of non-elective hospital admissions involve people with diabetes, with significant health and economic burden.

**Aim:**

To identify precipitating factors influencing diabetes-related unscheduled hospital admissions, exploring potential preventive strategies to reduce admissions.

**Design & setting:**

Thirty-six people with type 1 (*n* = 11) or type 2 (*n* = 25) diabetes were interviewed. They were admitted to hospital for unscheduled diabetes-related care across three hospitals in Scotland, Northern Ireland, and the Republic of Ireland. Participants were admitted for peripheral limb complications (*n* = 17), hypoglycaemia (*n* = 5), hyperglycaemia (*n* = 6), or for comorbidities presenting with erratic blood glucose levels (*n* = 8).

**Method:**

Factors precipitating admissions were examined using framework analysis.

**Results:**

Three aspects of care influenced unscheduled admissions: perceived inadequate knowledge of diabetes complications; restricted provision of care; and complexities in engagement with self-care and help-seeking. Limited specialist professional knowledge of diabetes by staff in primary and community care, alongside inadequate patient self-management knowledge, led to inappropriate treatment and significant delays. This was compounded by restricted provision of care, characterised by poor access to services — in time and proximity — and poor continuity of care. Complexities in patient engagement, help-seeking, and illness beliefs further complicated the progression to unscheduled admissions.

**Conclusion:**

Dedicated investment in primary care is needed to enhance provision of and access to services. There should be increased promotion and earlier diabetes specialist team involvement, alongside training and use of technology and telemedicine, to enhance existing care.

## How this fits in

Unscheduled care in the form of hospital admissions is a significant and potentially avoidable problem for people with diabetes. Unscheduled hospital admissions were influenced by various primary care factors, including perceived inadequate diabetes knowledge among staff, gaps in primary and community care management, and complexities in patient engagement. Inadequate community management was compounded by delayed access to diabetes specialist services, with specialist intervention often only provided following significant illness progression requiring admission. Dedicated investments are needed to enhance primary care knowledge and provision using technology to augment available services, promoting earlier intervention, and facilitating improved individual self-management and support.

## Introduction

Around 463 million people currently live with diabetes worldwide, which is anticipated to rise to 578 million within 10 years.^1^ Many people with diabetes do not achieve optimal glycaemic control, resulting in significant complications,^2^ which require unscheduled care. This is defined as non-elective health care such as emergency department attendance, ambulance callouts, out-of-hours care, and hospital admissions.

Approximately half of healthcare costs in people with long-term conditions, such as diabetes, are accounted for by unscheduled care.^3^ In the UK, diabetes accounts for approximately 10% of the entire health resource expenditure, 80% of which is attributed to preventable complications.^4^ Lower-limb amputations are one of the most devastating complications, with physical and psychosocial implications, alongside resource-intensive demand on health systems.^5^ Despite multiple initiatives to ‘put feet first’,^6–8^ peripheral limb complications are the predominant reason for acute diabetes admissions in Western countries,^8,9^ with 51.4% of UK diabetes-related hospital admissions owing to foot disease.^10^

Diabetes is an ambulatory care-sensitive condition, meaning that preventive primary care management should avoid unscheduled care.^11^ It is, however, the condition most highly associated with non-elective admissions, accounting for 18% of admissions,^12^ which highlights a stark need for approaches to avoid diabetes-related admissions. Pre-existing use of unscheduled care, lacking partner support, significant life events, depression, and poor primary care management are identified predictors of unscheduled care in long-term conditions.^3,12,13^ Continuity of care, enhanced primary care, and better access has been shown to reduce unscheduled care in people with diabetes.^14,15^ Unscheduled care has been shown to arise from a ‘pressing need’, as a ‘reluctant last resort’, influenced by previous experiences, perceptions of expertise in care provision, and service accessibility.^3,16^

Traditional healthcare models place specialist diabetes knowledge in secondary or acute care, with long-standing policy history to strengthen services outside of hospitals and treat people closer to home.^17^ In 2013, UK guidance on admission avoidance in diabetes recommended a whole-system approach to improve service delivery, which involves increasing patient support, education, and resources for primary care management of foot ulceration.^8^ Further recommendations emphasised a shift from acute to primary and community care^18^ to reduce pressure on unscheduled care services, emphasising preventive care and access to multidisciplinary foot care teams and specialist diabetes nurses.^19^

This study, as part of a wider programme to develop interventions to reduce diabetes-related unscheduled care, aimed to identify precipitating factors influencing diabetes-specific unscheduled hospital admissions, to explore potential preventive strategies.

## Method

Adults (aged >18 years) with diabetes (type 1 or 2) admitted to hospital for diabetes-related unscheduled care were identified by inpatient diabetes nurses. The nurses notified the study researchers, who then verified potential eligibility with ward staff before approaching individuals. Exclusion criteria were: elective admissions and non-diabetes-related admissions; inability to give written informed consent; unable to participate in an interview conducted in English; and/or too unwell to take part.

Demographic data were collected at recruitment and during interviews. Semi-structured interviews were conducted by three researchers using a topic guide, prompt sheet, and data collection proformas to ensure quality consistency. Interviews were conducted as close to the discharge date as possible (81% <4 weeks) and digitally audiorecorded with permission (one declination). Recordings were transcribed verbatim, checked for accuracy, and fully anonymised. Data on previous diabetes-related admissions and subsequent (re)admissions were collected retrospectively for NHS Highland (NHSH) in Scotland and Western Health and Social Care Trust (WHSCT) in Northern Ireland, these data were unavailable for Letterkenny University Hospital (LUH) in the Republic of Ireland.

Framework analysis^20^ was used to explore precipitating factors influencing unscheduled admissions, which is well suited to applied research with focused questions such as the current study. Analysis involves five key stages: 1) familiarisation; 2) identifying a thematic framework; 3) indexing; 4) charting; and 5) mapping and interpretation. Analysis was used iteratively, working within and across study sites as data were collected. Familiarisation and identification of preliminary thematic frameworks was conducted independently at each site, using a pre-defined code developed in relation to the study aims focusing on pre-admission factors. These were adapted following emergent findings before being shared across sites and consolidating the frameworks. Further refinement through charting, mapping, and interpretation was conducted by the first author in consultation with other authors to ensure agreement over the identification and analysis of emergent themes.

## Results

### Participants

Of 78 people expressing interest, 36 participants with type 1 (*n* = 11) or type 2 (*n* = 25) diabetes gave written informed consent and completed interviews. Forty-two were withdrawn owing to significant illness or impairment (*n* = 8), death (*n* = 1), change of mind (*n* = 1), or being uncontactable (*n* = 32).

**Figure 1.**
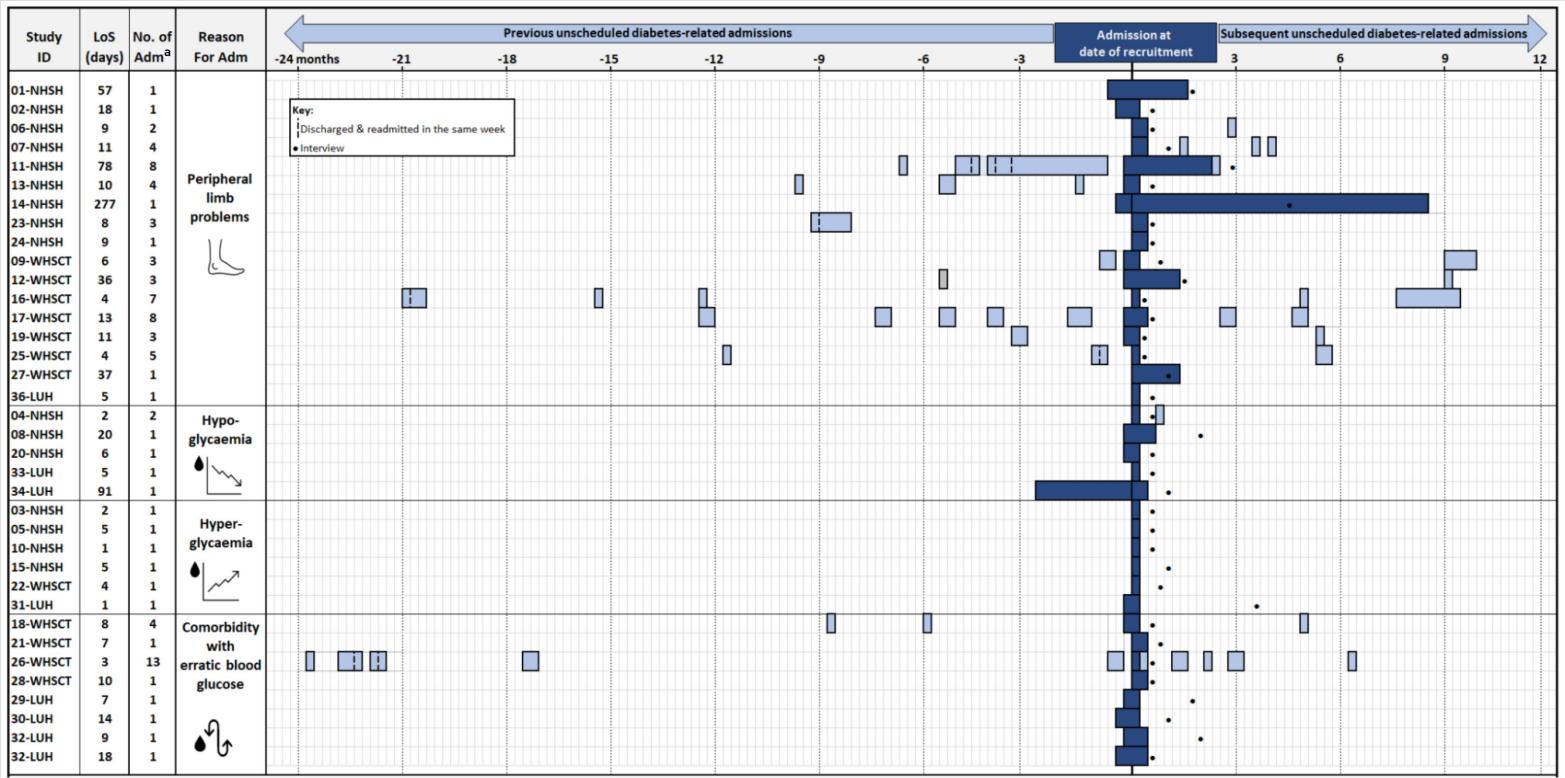
Overview of participant diabetes-related unscheduled admission history. ^a^Data for previous/subsequent admissions were not available for participants from LUH. Adm = admissions. LoS = length of stay (for admission at time of recruitment). LUH = Letterkenny University Hospital. NHSH = NHS Highland. WHSCT = Western Health and Social Care Trust.

Participant demographics and admission history are displayed in Table 1 and Figure 1. Participants were mostly male (*n* = 23), aged >50 years (*n* = 25), of White ethnic group, and identified as British (*n* = 15), Scottish (*n* = 10), Irish (*n* = 8), English (*n* = 2), or Scots-Canadian (*n* = 1). Seventeen were admitted for peripheral limb complications. Others were admitted for hypoglycaemia (*n* = 5), hyperglycaemia (*n* = 6), or comorbidities presenting with erratic blood glucose (*n* = 8). Length of stay varied considerably from 1–277 days (median = 8.5, interquartile range [IQR] = 5.0–13.5). Fourteen participants had previous or subsequent admissions, of which 13 were (re)admitted 6 months before and/or after their admission at recruitment. Five participants were discharged and readmitted within the same week on eight separate occasions, with more recurrent admissions seen in participants admitted for peripheral limb complications.

**Table 1.** Sample characteristics

	**All sites** (***n* = 36**)	**NHSH** (***n* = 16**)	**WHSCT** (***n* = 12**)	**LUH** (***n* = 8**)
Sex, *n*				
Female	13	8	3	2
Male	23	8	9	6
Age, years				
>60	17	11	5	1
50–59	8	1	4	3
40–49	8	4	2	2
30–39	2	—	1	1
<30	1	—	—	1
Type of diabetes, *n*				
Type 1	11	2	3	2
Type 2	25	14	9	6
Median duration of diabetes, years (IQR)	18 (11.5–28.5)	17 (10.5–26.0)^a^	17 (13.5–24.5)	25 (14.0–36.8)
Median distance from hospital, miles (IQR)	12.6 (3.7–17.9)	4.2 (1.75–14.6)^a^	10.4 (4.3–15.3)	22 (17.5–33.2)
Urban rural classification, *n* ^b,c,d^				
Large urban areas	5	—	5	—
Other urban areas	10	8	2	—
Accessible small towns	2	1	—	1
Remote small towns	3	—	—	3
Accessible rural	11	4	5	2
Remote rural	3	1	—	2
Very remote rural	1	1	—	—
Reasons for admission, *n*				
Peripheral limb complications	17	9	7	1
Hypoglycaemia	5	3	—	2
Hyperglycaemia	6	4	1	1
Comorbidity with erratic blood glucose	8	—	4	4
Day of admission, *n*				
Monday	11	7	3	1
Tuesday	6	2	4	—
Wednesday	5	3	2	—
Thursday	3	1	1	1
Friday	6	1	2	3
Saturday	5	2	—	3
Sunday	—	—	—	—
Median length of stay, days (IQR)	8.5 (5.0–13.5)	9.0 (5.0–18.5)	7.5 (4.0–11.5)	8.0 (5.0–15.0)

^a^One participant not reported. ^b^Based on Scottish Government Urban Rural Classification.^51^^c^No responses for ‘very remote small towns’. ^d^One participant did not report due to living in the hospital.

IQR = interquartile range. LUH = Letterkenny University Hospital. NHSH = NHS Highland. NR = not reported. WHSCT = Western Health and Social Care Trust.

### Findings

Three factors influenced diabetes-related unscheduled admissions: perceived inadequate knowledge of diabetes complications; restricted provision of care; and complexities in engagement with self-care and help-seeking.

#### Perceived inadequate knowledge of diabetes complications

When exploring participants' admission history, a reported lack of specialist knowledge in diabetes foot care was prominent, describing inadequate expertise in community services influencing the progression of illness leading to admissions. Several participants with peripheral limb complications discussed management by their general practice nurse, experiencing ineffective treatment and/or advice for their ulceration, for example:

*’I got an appointment with the practice nurse, who wasn’t used to — it wasn’t her specialty. By this time, the ulcer was quite painful, and I wasn’t really walking …her* [practice nurse] *view was take painkillers and just act normally.* [Practice nurse] *wasn’t too strong but, she* [podiatrist] *said, ”Stay off that foot.” And that was the best advice I had* *.’* (Identifier [ID]6/NHSH)

Although some participants experienced positive outcomes once appropriate treatment and guidance was received, others had to demand to be seen by specialist teams. In one case, delays meant that ulceration progressed to requiring surgical intervention, a narrative similarly recounted by three other participants. One example:

*’I had a foot ulcer on my left foot and all she done for four months was put bandages on it … Never a specialist seen … Then … she took a picture of it and showed me it and I says, ”You need to get me a doctor! I’m sick of putting bandages on it, it’s not doing any good.” Then I got a doctor … he looked at it and … I was out with him at half past one and lying on the operating table at half past four getting it amputated.’* (ID11/NHSH)

The lack of specialist knowledge described by patients closely aligned with the theme of restricted provision, with delays reaching a critical point; for example, several participants across the sites identified these two factors culminated resulting in admissions. Participants often described delays owing to inadequate primary care provision for foot problems. Once seen by appropriate specialist teams, immediate hospital referrals were made since progression was so severe they could no longer be treated in community services:

*’Well, I’ve had an ulcer on my heel for probably the best part of a year…And, it got very, very bad at one stage…Now, she* [podiatrist] *actually* *…* *she found a hole 3 cm deep. And she said, you’re in the wrong place, you would need to go to* [city]*. Now, today, right this minute.’* (ID19/WHSCT)

A sense of not being heard owing to inadequate professional knowledge was communicated by this participant. Despite repeatedly raising concern that their ulcer was inappropriately assumed to be healing, prolonged delays in being referred to podiatric services resulted in admission:

*’So, I was actually on* [antibiotics] *for nearly a month before the podiatrist discovered … every appointment they always told us,* *”* *Well, it’s getting better … it’s getting smaller.”* *But we kept saying,* *”* *But why’s the pain so terrible?”* *I was mentally drained, nobody took us on.’* (ID19/WHSCT)

Although specialist services were described as present across the study sites, access to this was often noted as limited, or appointments were only offered following admission. One person described a sense of inadequacy in their treatment because only after they presented at the emergency department, and admitted to hospital for 9 days to treat an infected toe, did they receive specialist diabetes nurse involvement in their care:

*’I haven’t had an awful lot of appointments…I just feel…maybe a wee bit more could have been done…More check-up appointments, more thoroughly…It’s just very basic…I have an appointment now with my diabetic nurse…because of all this.’* (ID24/NHSH)

This participant also described their own inadequate understanding of self-management combined with poor diabetes education, contributing to late help-seeking behaviour reaching a critical point requiring admission:

*’If I had actually been a bit quicker but…just thinking it was just a stubbed toe and it would get better…I have, for quite a while…been having a loss of feeling in my feet…* [Sighs] *I don't feel that I really know enough about it* [diabetes]*. Yes, there’s lots of literature out there but a lot of it’s double Dutch and it’s frightening* *.’* (ID24/NHSH)

Multiple participants discussed their own inadequate knowledge of diabetes self-management as a contributing factor in the progression to admission, which was more prominent in participants with higher frequency of admissions. Accounts described inabilities to self-monitor owing to requiring education and/or equipment. One participant discussed only being reviewed at annual general practice appointments, despite concerns being raised in the year before being hospitalised for severe hyperglycaemia:

*’My CPN* [community psychiatric nurse] *…* *he’s been saying for the past year that they should be checking my blood* *…* *But they didn’t* *…* *So, obviously it slowly got worse and worse* *…* *I can do it myself now* [following admission] *…* *But not before. No.’* (ID5/NHSH)

#### Restricted provision of care

Poor access to GP appointments and insufficient out-of-hours services were factors identified as influencing admissions. More admissions were seen around weekends (Friday to Monday), with one interviewee describing unavailable out-of-hours support, meaning that adequate glucose control could not be achieved. Once seen that Monday, symptom progression meant that specialist intervention was needed:

*’So, that Monday morning, because she’d had a bad weekend, community nurse said,”Bang, right straight to* [hospital]*”* *…* *it’s quite a tricky act, you know?’* (ID3/NHSH)

Several participants described profound difficulties in obtaining a GP appointment, with some recounting a subsequent use of out-of-hours services. One person acknowledged that the use of out-of-hours services often resulted in hospital attendance:

*’Aye, it is really hard to get an appointment* [with GP]*. You have to ring weeks in advance* *…* *I have used the out* *of* *hours* *…* *it usually leads to you going into hospital* *.’* (ID26/WHSCT)

Multiple accounts also described poor continuity of care and a sense of futility in not having a regular point of contact:

*’So, I goes to make an appointment because of this…One month before she could see me, and I said,”Who’s my doctor now?”, because you see when you go down, you don’t get the same person. All the time.’* (ID27/WHSCT)

The influence of restricted provision on admissions was further compounded by proximity to services for participants in remote and rural locations. These accounts described deficient local support, with services often centralised to the ‘main hub’ of towns and cities. Some participants also described a reliance on limited public transport, resulting in further delays and inadequate follow-on care post-admission:

*’I still haven’t seen the diabetic nurse in my area yet…I’m supposed to be waiting for her so she can give me more information…I can get the train, but I’d have to start out at ten, and then I probably wouldn’t get home until eight-thirty… it’s mainly in* [city]*. But it’s hard to get things round here* *…* *It’s quite difficult to get help* *.’* (ID5/NHSH)

Rurality was also discussed as exacerbating potential admission owing to the delays in receiving necessary care. One participant discussed the potential for already serious issues becoming aggravated by the time taken to travel to or from remote and rural locations:

*’* [Town] *is your main hub, if you have an emergency it’s going to be, by the nature of it, a bigger issue with the distance that you have to travel in time* *.’* (ID33/LUH)

#### Complexities in engagement with self-care and help-seeking

Nearly all participants described challenges in self-care influencing illness progression, although many described this as historic, feeling well equipped to effectively self-manage now. Exploring participants' help-seeking before admission revealed avoidance driven by fear of services and accountability. One participant described disengagement from their diabetes specialist nurse (DSN) coupled with an acknowledgement that once seen, owing to illness progression requiring specialist intervention, that this was more reassuring than they had anticipated:

*’The community diabetic nurse…it’s easier to hide from them than it is to go to them. Once they suck you in and nail you down it’s actually not so scary…It’s just getting over that initial hurdle of speaking to professional people without fearing them…I think it’s getting into the mindset of being accountable.’* (ID4/NHSH)

This was echoed by another participant who openly discussed disengaging from diabetes specialist teams owing to the burden of their condition:

*’I’m probably the opposite, I maybe disengage from them a bit sometimes…it just becomes overwhelming… I never let it on that I’m unwell. I just don’t like feeling vulnerable.’* (ID7/NHSH)

This theme was further developed by participants discussing difficulties in accepting the severity of their illness, so help-seeking was not undertaken until necessitating admission. This was prominent in participants with peripheral limb complications; for example, one participant, who previously worked as a nurse, with sufficient knowledge of the risks and implications of their symptoms, described their reluctance to acknowledge the gravity of the situation, resulting in amputation:

*’Well, I recognised there was something wrong, but I didn’t take action to do anything about it. As a nurse, I knew there was something wrong…I ignored it, hoping it would go away…I should’ve taken action right away. I mean a lot of people who weren’t nurses would have gone right away. I didn’t.’* (ID2/NHSH)

This participant had also discussed a previous admission for ulceration where amputation had been avoided, highlighting the complexity in engaging in self-care and late help-seeking despite adequate knowledge and experience of the risks and consequences:

*’A few years ago I was in when my foot was really, really bad. I was in for a while…What work they put in…they saved my toes, there’s no doubt about it…well, up until now anyway.’* (ID2/NHSH)

Nearly all participants with peripheral limb complications recounted either inaction or significantly delayed responses to experiencing peripheral neuropathy, a strong antecedent to ulceration. One participant stated that they ignored the symptoms for many years, not establishing a sense of urgency in their illness beliefs until pain was experienced, by which point admission was required:

*’I started losing feeling to my feet…Years ago…But there was still no pain so I never bothered about it.’* (ID16/WHSCT)

## Discussion

### Summary

The three compounding themes — perceived inadequate knowledge of diabetes complications; restricted provision of care; and complexities in engagement with self-care and help-seeking — exposed a complex multifaceted issue when exploring diabetes-related unscheduled admissions. Perceived inadequate primary care specialist diabetes knowledge was closely interwoven with restricted provision, characterised by limited appointments and/or proximity to services, poor continuity of care, and significant delays. This was further impacted by complexities in participant engagement with self-care, help-seeking, and illness beliefs resulting in progression to admissions.

### Strengths and limitations

The findings offer novel insight into precipitating factors for admissions, corroborating and building on existing evidence on unscheduled care. All participants were of White ethnic group; while reflecting the local demographics of the study locations, this meant that experiences from minority ethnic groups was lacking. The findings are, nonetheless, considered representative of patients requiring diabetes-related unscheduled care in rural areas, since analyses identified common interwoven elements across the sites, despite data being collected from three countries. Owing to delays in site-specific ethical approval, the data collection for sites was staggered, influencing the number of participants recruited. Data collection at LUH was approved after 8 months from the first approval, resulting in a smaller sample and unattainable admission data. Thirty-two participants did not respond to communication following discharge. Although it was considered likely that these patients were too unwell to respond, this could not be confirmed, which could suggest a source of selection bias. The data collection only reflected the experience and perceptions of people with diabetes without representative data from healthcare professionals (HCPs) in the study sites. Although this fell outside the scope of the study, this would have provided a more fully informed analysis.

### Comparison with existing literature

The findings extend existing understanding that previous experience of unscheduled care, primary care management, poor continuity of care, and psychological factors influence unscheduled care in diabetes.^3,13–16^

Despite existing health policies recommending delivery of care in primary and community services,^8,18,19^ a perceived lack of specialist diabetes knowledge presented as a factor in participants’ admissions. Inadequate specialist primary and community care knowledge resulted in insufficient treatment, particularly in foot care, and/or delays resulting in hospital attendance. It has long been stressed that HCPs managing diabetic feet in primary care must be adequately trained to do so.^7^ Nonetheless, insufficient priority is given to basic and continued training for medical professionals,^21^ with recent UK national audits identifying 32% of commissioners not providing foot examination training to primary care staff, and 39% of people with an ulcer having to wait >2 weeks for their first specialist appointment.^22^ While participants with peripheral limb complications did discuss specialist team involvement, this was often successive to significant delays, despite evidence suggesting that rapid access to specialist teams can significantly reduce hospital intervention.^23^ Such delays are seen across the UK and Europe,^24^ with a recent meta-analysis suggesting poor symptom recognition, inaccurate assessment, and limited access to specialist services as frequent reasons,^25^ echoing the current findings. While existing guidelines set out care pathways for referral between primary and specialty services,^26,27^ there was considerable variation in the recommendations across the study sites. A lack of clarity and acceptance on fundamental components of care suggests that clinical decisions can be influenced by judgement rather than standardised support tools.^21^

Specialist knowledge was closely aligned with difficulties in provision, with varied accounts describing a compounding interplay between inadequate HCP knowledge, rurality, limited access to appointments, and poor continuity of care culminating in significant delays leading to admissions. Many participants described approaching their GP with diabetes-related concerns, with limited or no access to a DSN, despite being recommended as the first point of contact for people with diabetes.^28^ A specialist nursing taskforce survey highlighted significant crisis in DSN workforces, acknowledging that those who cover large demographic rural areas, pertinent to the current study, often lack the additional support required for their caseload.^29^ This substantiates research that has shown people with diabetes and chronic complications living in remote locations experience poorer continuity and do not receive the recommended specialist care required, subsequently experiencing adverse health outcomes.^30^ The limited access and continuity described by participants amalgamated with a strong desire for a consistent point of contact to facilitate their health. The salience of this is amplified when coupled with research showing that continuity of care significantly improves outcomes, and reduces admissions and mortality.^15,31^

Further to HCP specialist knowledge and understanding, it was clear from the current findings that multiple participants struggled to adequately self-monitor, both in terms of their blood glucose and/or their foot care. Although some access to self-management education was discussed, this was often retrospective to urgent care rather than preventive management. Appropriate education is a vital care component for people with diabetes, yet adequate utilisation and delivery remains a challenge, although use of technology-enabled models shows promise in increasing reach and engagement.^32^ Many participants with peripheral limb complications described previous experiences with ulceration and/or amputation, corroborating research that the strongest predictor of ulceration is having a previous foot ulcer.^33^ Patient education has been shown to be effective in reducing peripheral limb complications, but without continuation beyond initial standalone sessions this does not effectively prevent ulcer recurrence.^34^

Complexity in participants’ illness beliefs and health-seeking behaviour, particularly in those with peripheral limb complications, stood as a final compounding factor influencing unscheduled admissions. Research has shown that the illness beliefs around diabetic foot ulceration, specifically understanding of and perceived experience and control over ulceration, independently predict health behaviours.^35^ Variance in self-care behaviours has been shown to be far greater in people with peripheral limb complications when exploring the effects of illness beliefs on other self-care behaviours in diabetes.^35,36^ This supports the current findings where participants with existing ulceration and/or amputation demonstrated difficulty in reconciling their experiences with their perceptions and subsequent health behaviours. Although not specifically recorded, multiple participants discussed comorbid depression and psychological difficulties, which is supported by research showing strong associations between foot ulceration and depression.^37–40^ This suggests a need for not only improved education, but also increased psychosocial support. In doing so it would encourage acknowledgement of illness severity, improve understanding of timely preventive self-care practice, and enhance engagement with services at appropriate junctures to avoid the progression of illness to requiring admissions. It has been acknowledged that behaviour change strategies in people with diabetes need to move beyond simply providing education, to ensuring adequate frequency of feedback with active guidance and support, problem-solving, and screening for psychological factors.^41,42^ The lack of continuity described by participants raises the question of who exactly should provide such strategies in a realistic and achievable approach.

### Implications for practice

The findings highlight a complex interplay of factors that would not be efficaciously managed by targeting each component alone, with a clear need for standardisation of guidance in assessing diabetes complications, especially diabetes foot care. Although significant recent progress has been made to standardise care,^43^ there is a need to ensure that resultant training extends to all relevant staff. As shown in the current findings, the multidisciplinary landscape of diabetes care delivery means that it is not always GPs who identify complications in primary care, but nurses or other primary care HCPs, which can lead to delays and/or inadequate provision.^24^

Put simply, the outcomes suggest a need for greater involvement of diabetes specialist teams within primary and community care services and suitably prepared DSNs and podiatrists, with adequate expertise and knowledge to provide necessary specialist support and increased continuity. Feasibility of such increased provision, however, is unlikely in the current health climate, with an even greater need to keep people out of hospital and provide care remotely, where possible, owing to COVID-19. Delivering adequate care could, however, be facilitated by technology. For example, it could be used to provide training for primary and community staff, enhance clinical decision support between staff and specialist teams, and improve continuity, education, and support for patients.

In the last year, COVID-19 has presented a need for greater investment and reliance on technology and telehealth to support patients with diabetes.^44^ Technology has the potential to enhance care through bidirectional communication, analysis of monitoring data, customising education, and individualising care plans.^45^ Lacking a regular point of contact for advice, support, and guidance was a prominent factor in the current findings. Technology could provide greater continuity alongside earlier referral to a DSN by providing more immediate and regular support, with a consistent point of contact to improve engagement and avoid unscheduled admissions. Telehealth has shown promise through handheld communication devices and interactive online systems. It provides immediate assistance and support for people with diabetes,^46^ with remote monitoring technologies reducing risk of adverse health outcomes.^47^ The feasibility, reliability, and validity of telehealth specific to diabetic foot complication has recently been shown to be effective, although cost efficiency is still to be demonstrated.^48^ Recent meta-analyses showed that telehealth for rural locations improved glycaemic control, patient empowerment, and self-management education,^49^ with telemedicine in diabetes found to be more effective than usual care.^50^ Incorporating data monitoring into decision making not only individualises care delivery, but also has been shown to improve engagement and effective behaviour change.^32^

There is a pressing need for specialist primary and community care diabetes provision, emphasising preventive strategies that can respond to the multidimensional risks identified in the current findings. The results call for increased investment and provision for specialist teams to facilitate support and education, with enhanced technology to bolster remote care delivery.
